# A qualitative study examining the benefits and challenges of incorporating patient-reported outcome substance use and mental health questionnaires into clinical practice to improve outcomes on the HIV care continuum

**DOI:** 10.1186/s12913-018-3203-x

**Published:** 2018-06-07

**Authors:** Anne K. Monroe, Sarah M. Jabour, Sebastian Peña, Jeanne C. Keruly, Richard D. Moore, Geetanjali Chander, Kristin A. Riekert

**Affiliations:** 1 Department of Epidemiology and Biostatistics, Milken Institute School of Public Health, George Washington University, 950 New Hampshire Avenue, NW, 5th Floor, Room 507, Washington, 20052 USA; 2 Division of General Internal Medicine, Johns Hopkins University School of Medicine, Baltimore, USA; 3 Division of Pulmonary and Critical Care Medicine, Johns Hopkins University School of Medicine, Baltimore, USA

**Keywords:** Patient-reported outcomes, Mental health, Substance use, Retention, HIV care

## Abstract

**Background:**

Inadequate identification and treatment of substance use (SU) and mental health (MH) disorders hinders retention in HIV care. The objective of this study was to elicit stakeholder input on integration of SU/MH screening using computer-assisted patient-reported outcomes (PROs) into clinical practice.

**Methods:**

We conducted semi-structured interviews with HIV-positive patients who self-reported SU/MH symptoms on a computer-assisted PROs (*n* = 19) and HIV primary care providers (*n* = 11) recruited from an urban academic HIV clinic. Interviews were audio-recorded and transcribed. We iteratively developed codes and organized key themes using editing style analysis.

**Results:**

Two themes emerged: (1) Honest Disclosure: Some providers felt PROs might improve SU/MH disclosure; more were concerned that patients would not respond honestly if their provider saw the results. Patients were also divided, stating PROs could help overcome stigma but that it could be harder to disclose SU/MH to a computer versus a live person. (2) Added Value in the Clinical Encounter: Most providers felt PROs would fill a practice gap. Patients had concerns regarding confidentiality but indicated PROs would help providers take better care of them.

**Conclusions:**

Both patients and providers indicated that PROs are potentially useful clinical tools to improve detection of SU/MH. However, patients and providers expressed conflicting viewpoints about disclosure of SU/MH using computerized PROs. Future studies implementing PROs screening interventions must assess concerns over confidentiality and honest disclosure of SU/MH to understand the effectiveness of PROs as a clinical tool. More research is also needed on patient-centered integration of the results of PROs in HIV care.

**Electronic supplementary material:**

The online version of this article (10.1186/s12913-018-3203-x) contains supplementary material, which is available to authorized users.

## Background

The HIV treatment continuum includes steps to achieve and maintain HIV suppression, and reduce HIV transmission. These steps include linkage to and retention in HIV care through regular HIV clinic visits, antiretroviral therapy (ART) prescription, ART adherence, and HIV RNA suppression [1–3]. Optimal engagement in HIV care allows people living with HIV (PLWH) to benefit from HIV therapy to improve their own clinical outcomes and to prevent the transmission of HIV to others [3]. However, retention in the United States is low, with over 50% of diagnosed persons failing to establish or remain engaged in medical care and less than 25% of all PLWH stably suppressed on ART [3].

Substance use (SU) and mental health (MH) disorders are highly prevalent among PLWH and are associated with worse HIV outcomes. The prevalence of SU among PLWH is high, reported in 10–40% of PLWH [4–7]. The lifetime prevalence of depression among PLWH has been reported as high as 45% [8] with recent depressive symptoms endorsed by 20–50% of PLWH [9–12]. Dual diagnosis is common; a large prevalence study demonstrated that 38% of PLWH had both a SU and a mood disorder [13]. SU and MH disorders among PLWH are associated with worse adherence to medication, appointment attendance, and other self-care behaviors [7, 14–16]. Previous studies have revealed an association between these disorders and worse retention in care [17, 18].

Furthermore, there is inadequate treatment of SU/MH among PLWH, starting with the under-recognition of these disorders. Among PLWH with SU disorders, both discussions with providers regarding SU and receipt of SU disorder treatment are low [19]. Prior research has demonstrated that effective SU/MH disorder treatment can improve HIV clinical outcomes [20–23]. However, as in general primary care, depression is under-recognized [24] and goes untreated or undertreated in many PLWH [25].

Computer-assisted interviewing may lead to increased patient disclosure and provider recognition of SU/MH disorders. Standardized screening using patient reported outcomes (PROs), defined as reports that come directly from the patient, rather than typical clinical care or chart review, increase identification of MH disorders [26]. There are many potential benefits of computer-assisted screening to increase patient reporting of SU/MH disorders. Routine use of patient reporting may enhance patient-provider communication. Furthermore, it may increase provider recognition of these problems and appropriate treatment and/or referral. Computer-assisted screening has been shown to have high acceptability in HIV clinics [27, 28]. However, potential barriers to clinical use of these screening measures include clinic space considerations, increased staff burden, and competing priorities for both patients and providers. To increase chances of success, PROs must be integrated into clinic flow in a patient- and provider-friendly manner, with high-perceived value of the information to providers [29].

The objective of this qualitative study was to assess the acceptability of using computer-assisted PROs by providers and patients in a clinic setting to facilitate identification and treatment of SU/MH disorders in HIV primary care. We were interested in learning if the perception of PROs differed by retention status. Since people who are not retained are more likely to have SU/MH issues, we were interested in whether they would be more or less likely to perceive use of PROs to identify SU/MH issues as helpful. Our long-term goal with this work is to determine if the use of computer-assisted PROs will improve outcomes along the HIV care continuum by improving engagement in care and management of SU/MH disorders.

## Methods

This study was nested within the Johns Hopkins HIV Clinical Cohort (JHHCC), an observational study of individuals receiving HIV care at the Johns Hopkins HIV Clinic in Baltimore, MD. The cohort has been described in detail previously [30]. Currently, JHHCC participants are offered computer-assisted PROs questionnaires every 6 months for research purposes and the results are not conveyed to the clinical team. These questionnaires include the Alcohol Use Disorders Identification Test Alcohol Consumption questions (AUDIT-C), the Alcohol, Smoking and Substance Involvement Screening Test (ASSIST), the Personal Health Questionnaire Depression Scale (PHQ-8), and the Generalized Anxiety Disorder (GAD-7) scale. At that time, no other SU/MH screening was routinely performed in clinic. In addition to those measures, the PROs include measures of adherence to HIV therapy, quality of life, etc. (full list in Interview Guide in Additional file 1).

From January to June 2016, we conducted 11 interviews with providers and 19 interviews with HIV-positive participants (hereafter referred to as patients). Of the 30 interviews, 29 were audio-recorded and the other interview (an individual living with HIV) was documented with field notes, because the patient declined to be recorded. The study sample was recruited as follows:

### Patient participants

English-speaking adult (≥ 18 years old) patients enrolled in the JHHCC, who completed a JHHCC PROs assessment from September 2013 through October 2014, and self-reported drug use, heavy alcohol use, or MH (depression or anxiety) symptoms using clinically relevant cutoffs from the screening tools were eligible for this study. This date range of PROs was selected to ensure at least 12 months of follow-up data was available to calculate retention measures. Retention status was calculated using the visit adherence measure (the proportion of kept visits/ (scheduled visits + kept visits)). Participants with a visit adherence score of less than 0.80 were considered not retained in HIV care. Medical record numbers from this roster of patients were matched with prior PROs for JHHCC participants who agreed to be contacted for other research and were subsequently sent a letter in the mail or approached to participate during a clinical encounter. We purposively sampled to obtain a mixture of patients who were retained and not retained and had SU only, MH symptoms only, or both.

### Provider participants

Clinic personnel, including physicians, nurse practitioners and physician assistants, from the Johns Hopkins HIV clinic were interviewed. Clinical personnel were sent an email from the principal investigator (PI) to describe the study and opt-in to participate in the study.

Both provider and patient participants signed informed consent; patients received $15 for their participation and providers received $25. This study was approved by the Institutional Review Board at the Johns Hopkins University School of Medicine.

#### Study development and design

The semi-structured interview guide was developed based on our conceptual framework, which incorporates factors influencing recognition and response to SU/MH disorders among providers, engagement in SU/MH care among patients in HIV primary care, and implementation of PROs as a clinical tool. The interview guide was iteratively revised as the study progressed. Patient interviews included questions regarding how SU/MH disorders affected retention in HIV care, prior attempts to access SU/MH treatment services, and perceived benefits and harms of using computerized PROs in clinical care, and preferences regarding receipt of PROs results by their own medical provider/other members of the clinic team. Provider interviews included questions regarding perceived benefits and harms of SU/MH treatment as related to retention in HIV care, provider/clinic obligation in response to positive screening for SU/MH disorders, preferences for incorporating computer-assisted PROs administration into the clinical workflow, delivery of PROs results. We asked questions regarding additional domains collected in the PROs, including sexual risk behavior and adherence to ART, however, this analysis includes only SU/MH data. (See Additional file 1 for Interview Guides).

Semi-structured in-person interviews were conducted by a trained research assistant for patient interviews and by the PI for provider interviews. The PI conducted the clinical personnel interviews, because the PI is familiar with the clinic flow and procedures needed to probe and obtain feedback from providers. Interviews lasted 15–20 min (patients) or 20–45 min (providers). Interviews were digitally audio-recorded and transcribed verbatim. No personal identifiers were included in the transcripts.

#### Data analysis

Using an application of grounded theory [31], the research team identified preliminary themes while reviewing the transcripts, and used an iterative consensus process to continue to refine themes as data analysis continued. Two separate comprehensive codebooks, (patients and providers) were developed based on themes. The team determined clear and applicable code definitions through an iterative consensus process. Two coders independently coded each transcript and came to consensus on discrepancies via discussion.

We did not formally calculate inter-coder reliability, rather we used established methods for addressing differences in coding due to multiple coders by addressing all coding discrepancies and reconciling them through discussion and consensus [32, 33]. We used QSR NVivo Version 11.0 to apply codes to the transcripts and organize text segments and relevant quotes abstracted during analysis. Editing style analysis, by which the coded text is organized to develop the final themes [34]. We examined whether different themes were endorsed by individuals who were or were not retained in HIV care.

## Results

### Demographics

The demographic characteristics of the patient sample categorized by retention status are summarized in Table 1. The racial and gender makeup of the sample were similar to the Johns Hopkins HIV clinic population, which is about 75% African American and 34% female. Overall, 57.9% of the sample was categorized as retained in HIV care. Twelve patients had active or recent SU, 4 had only MH symptoms, and 3 had both active/recent SU and MH symptoms. Although the sample size was small, we observed more non-retained participants with positive screens for both SU and MH. Additionally, we observed a higher number of overall positive screens among non-retained participants.

**Table 1:** Patient Sample Characteristics

	Retained (*N* = 11)	Not Retained (*N* = 8)
African American	11	8
Female	4	4
Age, mean (SD)	51.0 (6.9)	52.6 (4.7)
Age range	(33–61)	(46–61)
Positive on at least 1 SU screen; All MH screens negative	7	5
Positive on at least 1 MH screen; All SU screens negative	3	1
Positive on at least 1 MH and 1 SU screen	1	2
One screen positive (Either SU or MH)	9	3
Two screens positive (Either SU or MH)	1	4
Three screens positive (Either SU or MH)	1	1

The majority of the provider sample was female (*n* = 8, 72.7%). The sample included an array of health professionals, including 5 physicians (45.5%), 4 nurse practitioners (36.4%), and 2 physician assistants (18.2%).

Overall, there was some ambivalence about whether the added benefit of the computer-assisted PROs was worth the added effort on the part of the patients in completing the PROs and on the part of providers in reviewing them. Two themes that emerged were: (1) Honest Disclosure and (2) Added Value in the Clinical Encounter. We present these themes with illustrative quotations below and also provide tables (Tables 2, 3, 4) with additional quotations.

**Table 2:** PROs and Honest Disclosure of SU/MH – Provider Viewpoints

Qualitative Theme	Exemplar Quotation(s)
Less Willing to Honestly Disclose SU/MH with PROs	
Patient does not want provider to know about stigmatized behavior	“[It] worries me that they’re going to change their answers about substance abuse, alcohol, and adherence. At least many of them I think, don’t want to displease their doctors or don’t want to share everything with their doctors in as much detail, so once you start...telling the patients you’re telling their doctors...it might change answers.” (Physician)
More Willing to Honestly Disclose SU/MH with PROs	
More honest with computer	“That’s my understanding is that the data seems to show that if people can self disclose and it’s with a computer, they’re more likely to be honest.” (Physician) “Because those are like substance abuse, depression, obviously and a lot of time, patients won’t tell you and they would probably be more frank in answering it on the system would definitely help.” (Physician).

**Table 3:** PROs and Honest Disclosure of SU/MH – Patient Viewpoints

Qualitative Theme	Exemplar Quotation(s)
Less Willing to Honestly Disclose SU/MH with PROs	
Harder to disclose to computer	“It takes a lot for a person to express how they feel when you can be face to face with somebody. Emotionally and physically...being face to face with her ... It’s easier.” (Patient 20, Not Retained, MH only)
Fear of Provider Judgement	“Other patients see [will start] missing their appointments because they ashamed of what the doctor seen. Especially when you bring it to their attention...They’ll start shying away from the clinic” (Patient, Retained, SU only).
More Willing to Honestly Disclose SU/MH with PROs	
Easier to disclose to computer	“That would give the doctor herself an extra little something that well, I can read this and maybe they don’t want to open to me, but they’ll open up to this. So I think that’d be real good.” (Patient 19, Retained, SU only)

**Table 4:** Added Value in the Clinical Encounter – Provider Viewpoints

Qualitative Theme	Exemplar Quotation(s)
Challenges of PROs in the Clinical Encounter	
Difficult to prioritize	“What would be the hierarchy in what do we choose to address during this particular patient encounter versus what would we delay ... what is your obligation, then, to address everything, which I think is kind of a bigger issue if these are sort of fed back, do you have to address everything?” (Physician)
Detracts from patient-centeredness	“If I never ask those questions [referring to SU/MH questions] ...that would take away from that development of a [patient-provider] relationship, knowing who they are as a person and what it is that they’re dealing with.” (Physician) “I will only work in a room where I can share a screen with the patient so I want them to see what I’m seeing ...And I’m not sure how some are going to react [to PRO results]. I’m worried it could actually hurt the doctor patient relationship. One… [providers] may think they don’t have to ask because it’s been asked. Two, patients are going to see. Three, I’m checking boxes again.” (Physician)
Have information but patient may not be ready to do anything about it	“There is a potential I guess I think of it as little bit of analogous to a testing you know too sensitive in a way, which is that we’re going to screen more, we are going to find more. May or may not mean that the patients wanna do something about it.” (Physician Assistant)
Advantages of PROs in the Clinical Encounter	
Compelled to act	“I think we would all feel compelled to that oh, now I’ve seen this, I really need to do something about this.” (Physician)
Knowing severity helps set visit priorities	“I see severe I’m thinking, you know, we really need to spend a lot of time on this and if it’s moderate, I would give them a referral, but I think I might make different clinical positions depending on if I actually had an official screen for severity.” (Physician)
Springboard for discussion with patient	“You know, it’s kind of on the table and I can say you know, I’ve looked over your response forms and I’ve noticed these things, you know, what do you think? It looks like things are going well here, but maybe not here. You’ve talked...you haven’t told me about this recent drug use.” (Nurse Practitioner) “Look, we’ve gone through all of this stuff, you may or may not have talked to your doctor about it… Are there any of these you want to talk to your doctor about? Because then, it’s patient driven, it’s patient centered” (Physician). “And it just opens up the opportunity to again, talk about the availability of resources within the clinic to help better address that.” (Nurse Practitioner).
Improves efficiency	**“**It answers questions for me that I than frankly don’t have to initially ask.” (Physician)
Fills gap in practice	“So when I think standardization of questions that can summarize things for you in a very busy clinic where you have 30 min to get the evaluation done ...if it were done systematically at X intervals, for example, it would be very useful ... I see it as an opportunity to enhance my assessment of the patient and if ...there was something that was detected, then I could be more aggressive in approaching it” (Nurse Practitioner)
May capture issues that were previously undetected	“I think if you can actually pick up undiagnosed depression or undiagnosed anxiety and get someone into treatment for that, there’s actually a real value to actually having this screening and then being able to make the appropriate referral.” (Physician) “I think these questions are designed to try and tease out the people who are being over or under expressive on that [referring to the depression and anxiety standardized questionnaires]...I think sometimes those are harder questions as a clinician to ask” (Nurse Practitioner). “Having some other process...so picking up new problems, that’s very helpful, but when it’s an existing problem...You know I’m worried less about the extreme, which I think we pick up pretty well, but I think...it’s those in the middle where you know, they’re not clinically depressed, but they’re also not quite where they should be” (Physician).
Standardized questions	“I think it gives a more structured and quantitative way of looking at what people and what they are experiencing, so in that sense, I think it would be helpful.” (Physician)

### Qualitative themes

#### Theme 1: PROs and honest disclosure of SU/MH

Both providers and patients had mixed views about the PROs to improve detection of SU/MH issues and as a facilitator of honest SU/MH disclosure. There were opposing views on how forthcoming patients would be when disclosing to a computer compared with disclosing to a healthcare provider. Some providers and patients expressed that patients would be less honest if the results would be conveyed to providers; however, other stated they would be more honest.

#### Less willing to honestly disclose SU/MH with PROs

##### Challenges noted by providers

Some providers worried patients would answer differently if they knew their provider would see the PROs results versus when the PROs were completed solely as a research tool. Providers suggested patients would not want to disappoint their provider, so patients would perhaps be less willing to disclose SU/MH. Other providers expressed concerns that patients may be less forthcoming if their PROs results were documented in their chart:

*“I’m very much afraid their answers are going to change when they know it’s ‘part of their record’” (Physician).*

One provider also indicated that the patient-provider relationship fosters a safe space where patients may feel more comfortable being honest compared to the computerized PROs setting:

*“There are also people who...when they feel they can talk to their provider do talk to their provider pretty honestly when they know that they’re going to be able to speak...in a judgment free zone” (Physician Assistant).*

##### Challenges noted by patients

Like the providers, a few patients expressed hesitation to disclose honest results due to fear of being judged both in context of PROs and standard clinical encounters. One patient expressed that this fear of judgment might even drive people away from clinic and have a negative effect on the provider-patient relationship. Interestingly, none of the not retained patients expressed fear of provider judgment as a potential barrier to disclosing SU/MH problems. One patient described this fear of judgment in the following way:

*“A lot of times a lot of people [are] afraid to discuss it because then they may… then they thinking that now the doctor may have a different conception of me or view of me and start treating me differently” (Patient, Retained, MH only).*

Other patients suggested SU/MH disclosure would cause their provider to be disappointed. Social desirability could impede honest disclosure:

*“Certain things I wouldn’t disclose, because… I’ll feel bad to tell her that I’m drinking and missed my medication” (Patient, Retained, SU only).*

#### More willing to honestly disclose SU/MH with PROs

##### Advantages noted by providers

In contrast to the aforementioned viewpoints, some providers felt that the PROs as a clinical tool would improve patients’ ability to honestly disclose SU/MH. This would be beneficial to providers, because improved detection could lead to improved management. Rather than expressing concern about decreasing honest disclosure due to social desirability, providers expressed that the PROs would mitigate these fears, leading to more forthcoming disclosure:

“*There is a tendency for patients to down play the things that are really stigmatized with your provider...I don’t think that I get really good substance use history and understand what’s going on until, and this is an exaggeration, but until they are at the point where they really want to stop*” *(Physician Assistant).*

Many providers cited the literature indicating patients were more likely to disclose sensitive information to a computer than in a face-to-face clinical encounter. One provider expressed that the PROs would increase honesty, because the process would give patients the space and time to formulate truthful answers:

“*People sometimes don’t recognize what they’re thinking until maybe they’re having a quiet moment to read questions and think about their [answer]...maybe the written or the readable word helps people be a little more honest*” *(Nurse Practitioner).*

##### Advantages noted by patients

Like providers, patients felt the PROs would be beneficial, because it would provide a tool to help patient alert providers to issues that patients are unlikely to disclose during their appointment:

*“But if you see it on black and white… they [patients] won’t tell you, but they’ll put it on paper… [Interviewer asks why patient thinks this is] Shame. Shame. Shame” (Patient, Retained, SU only).*

Some patients indicated that provider recognition of honest disclosure on the PROs would foster improved rapport between patients and providers. This patient expressed his provider would appreciate forthcoming answers on the PROs results:

*“I think it would be about appreciation of honesty from the patient if my doctor was to read something and say oh wow. He was really honest about everything” (Patient, Retained, SU only).*

#### Theme 2: Added value in the clinical encounter

As noted by many clinicians, the busy clinical setting, with full schedules and a short time allotted for appointments, sometimes left providers stressed and unable to sufficiently and consistently assess for SU/MH problems. This was compounded by the varying needs that are a patient’s priority for the visit. Some providers felt the PROs would detract from patient-centered care, because it would be something else in the visit that needed to be addressed. Several providers felt they would have a difficult time prioritizing, planning, and carrying out a response personalized to each patient’s needs and that the additional information from the PROs might make that harder. Conversely, many providers expressed that the PROs results would alleviate the burden of screening for SU/MH. The PROs were seen as a detailed and structured assessment to alert and guide the provider to address the issue. Patients did not express concern about the potentially overwhelming volume of information the PROs would provide but did think the PROs would improve the care from their HIV provider, including SU/MH treatment.

##### Challenges noted by providers

Generally, providers were concerned that viewing all PROs results would take too much time to process and evaluate to effectively incorporate and prioritize for a clinic encounter. This overwhelming volume of information could potentially consume the clinical encounter. As one provider noted:

*“The only downside I see is ...that we might not be able to address anything or everything or necessarily know how to address everything” (Physician).*

Providers generally endorsed wanting a score for the assessment along with an interpretation and recommendation for the results. Some providers suggested incorporating the assessment results into the EMR; however, they worried that including the information in the EMR would add time without improving the patient-provider relationship or medical care. A minority of providers feared this technological-based intervention had the potential to decrease patient centeredness:

*“I’m worried that we spend less time doing what we should be doing, which is talking with our patients, examining them, explaining things, hearing their input, as opposed to looking through yet another series of things that I have to go through”(Physician).*

Another concern that was expressed by a minority of providers was that the SU and MH history taking in the patient interview is an integral part of building the patient-provider relationship, and that providers themselves should ask SU/MH questions rather than having this part of the history conducted by a computerized PRO.

##### Challenges noted by patients

A minority of patients expressed that the PROs would not be valuable to them personally as a clinical tool, because they already disclosed all issues to their provider and would not change the patient-provider relationship or the interaction at medical visits. These were patients with a strong relationship with their provider:

*“I don’t keep nothing from my doctor...I really don’t. You know, you keep stuff from your doctor, they can’t treat you” (Patient, Retained, SU only).*

One patient who described a close patient-provider relationship subsequently noted that other patients might not be as forthcoming with their doctor; it is unclear, however, if they felt that this problem will be ameliorated with the PROs approach.

Patients expressed concern about who would view their PROs results, stating that they would need to get to know the provider before they would be comfortable having their results disclosed. Some patients also described the lack of control over who could view the PROs results once transmitted to the medical chart. As the following patient said:

*“I don’t want everybody to know my business and like I said… for it to be confidential” (Patient, Not Retained, SU only).*

Although patients did not explicitly state that they would complete PROs differently, talking about SU/MH evoked concerns about confidentiality and trust, whether through verbally reporting problems or on the PRO. Patients described needing a foundation of trust in order to disclose issues:

*“For me to tell you about my private life and what’s going on with it, I have to like you and trust you” (Patient, Not Retained, SU only).*

##### Advantages noted by providers

While recognizing that PROs results might affect clinic flow, other providers were less concerned about the time to review the results and expressed that the PROs would both fill a gap in the provider’s practice by improving the assessment and detection of SU/ MH disorders and save time. For example, one provider expressed how the PROs would detect SU/MH problems that the provider is currently unable to consistently and thoroughly assess:

“*A lot of time in the visit, you just get backed up with whatever the patient brings in for the visit like that you don’t really get to ask all those questions” (Physician).*

The detailed and standardized PROs results were seen as beneficial to provider practices by improving detection of SU/MH disorders, as the following provider expressed about the depression-screening questionnaire:

“*I do not screen, I don’t ask these eight or ten questions... I believe, and it’s all it is a subjective belief, I believe I’m pretty good at picking up on it, but I have no proof*” (Physician Assistant).

Additionally, several providers stated that knowing the severity of a patient’s issue based on the PROs results could help them prioritize what to address at the visit.

Two providers presented implementation strategies to ensure the PROs can be used to supplement and support the HIV primary care visit rather than detract from patient-centered care. One physician suggested:

“*What if the patient does the PROs and they get the feedback, gosh, you have high scores on depression, drug use, and low physical activity, which of these would you like to talk to your doctor about today?” (Physician).*

A few providers indicated the PROs results could be seen like a lab value, to which prompt evaluative action on the part of the provider:

*“The same way if you have a CT scan with an abnormal cancer and we get this information. This is important. This could be a barrier to patient’s treatment” (Physician).*

Finally, in the discussion of the PROs at the study site, where they are already in use in a research capacity, one provider expressed the importance of research results that provide important information for clinical diagnoses and management and expressed frustration at not receiving the results:

*“You guys do get this information every six months whether I get it or not. What do you guys do with it? [Response from interviewer: It goes into the research database]. That’s the same thing those research people do. They get stuff and they don’t tell you anything” (Physician).*

##### Advantages noted by patients

Patients expressed the PROs could improve the patient-provider relationship by increasing the “bond” with providers. Patients felt the PROs would improve the care they received, because the results would be valuable for treatment management:

*“They would go the…extra mile to help me more, to try to take another step … See, see I’m getting better, get off the drugs and stuff” (Patient, Not Retained, SU only).*

Because of the perceived value for improving care, a patient was frustrated that research results were not used to improve care and stated:

*“Even though we take them kind of surveys and they ask you them kind of questions. I still don’t see nowhere where they trying to assist someone who do have a problem…So why asking me? When you asking them questions, you just really being nosy because ...nothing been implemented to help people that is using that really may want to get off using” (Patient 18, Retained, MH only).*

This quotation suggests that some patients complete surveys with the hope that the responses will trigger support, even if they have been told it is only for research.

## Discussion

In a qualitative study of providers and PLWH from an urban academic HIV clinic, stakeholders expressed optimism about the potential for computer-assisted PROs to enhance identification of SU/MH disorders. The main themes identified about the use of computerized PROs in clinical care were: 1) Honest Disclosure and 2) Added Value in the Clinical Encounter. Patients and providers had mixed views about the impact the computer-assisted PROs would have on honest disclosure. One potential benefit was increased honesty when interfacing with a computer-delivered questionnaire, as there would be less social desirability bias as compared to a provider interview [35, 36]. However, the opposing view was that the strength of the patient-provider relationship ultimately determines whether patients disclose SU/MH issues to their providers and that the computer-assisted PROs might actually decrease patient-centeredness and rapport building. Some providers expressed concern regarding impeding clinic flow due to PROs, with others stating that having the results of SU/MH assessments could actually expedite care. Prior work has shown that screening for substance use [37] and mental health [38] in a primary care setting is acceptable to patients. However, time was cited as a barrier to implementing screening and brief intervention [39], which was raised by some providers. Patients were not certain that PROs would augment the clinic visit if their provider “already knows everything” about them.

The ultimate impact of computer-assisted PROs on retention in HIV care is unclear. Prior to starting the interviews, we thought there might be different views of PROs expressed by retained and not retained patients (e.g., non-retained patients might express more concerns about confidentiality or limited usefulness of the PROs). However, patients’ views on PROs did not vary substantially by retention status. This suggests that PROs completion itself is likely not a barrier to retention, but beyond that conclusion, it is difficult to predict the effect of PROs on retention without additional research. What became clear during the study was that the integration of PROs must reinforce patient-centeredness and strong patient-provider relationships. This will foster an environment where the important work to ensure adequate follow up of a newly identified SU/MH disorder can occur.

There is long-standing interest in using PROs to improve detection of clinical issues, improve patient-provider communication, and monitor response to treatment [40], however, there is still little empiric data supporting the clinical utility of PROs [41]. The results of a qualitative study of providers in HIV clinic sites that have integrated PROs into clinical care [42] differed from ours in that those providers did not report concern that the patients might fail to disclose behavior on the PROs. At those clinics, the results are viewed by the provider but not entered into the patients’ medical records. Similar to our results, the authors noted that providers who had confidence in the strength of their relationship with the patient were less likely to find the PROs helpful. It has been previously shown that self-administration (compared with face-to-face interview) may increase the disclosure of stigmatized conditions or behaviors [43, 44]. However, providers still may not trust that patients are truthfully disclosing [45]. From the patient side, at least one patient highlighted the need for a robust rapport between patient and provider to improve detection and management of SU/MH issues. Without this foundation patients may be unwilling to disclose such issues in the clinic setting. Additional findings pertinent to our future work were that providers wanted patients to identify an issue that was pressing to them, rather than simply receiving results of all screens. Finally, providers wanted more training on managing the results of positive screens. Our work adds to the published literature by eliciting viewpoints from both patients and providers and starting to examine the impact of PROs on retention in HIV care.

The limitations of our study are that we had data from one clinic, and we were recruiting individuals who had already agreed to participate in the PROs administered through the parent cohort study; we did not have information from people who refused the PROs even as research tool. The SU/MH symptom data and subsequent retention data was collected in advance of the interview for this study and may not have accurately reflected current behavior of the PLWH in care. Finally, we did not recruit anyone who was completely disengaged from care, i.e., not attending HIV clinic, therefore our responses may not accurately reflect their concerns and viewpoints. A potential limitation of the provider interviews, given that they were conducted by the Principal Investigator, was that colleagues who knew the Principal Investigator’s line of research might not have been as forthcoming about negative perceptions about the use of PROs.

## Conclusions

With this work, we gathered stakeholder input regarding logistical challenges of implementing SU/MH screening in a busy clinic as well as the perceived utility of screening. Additionally, we heard from patients about their experiences disclosing SU/MH in their HIV primary care appointments. Patients believed that disclosure can help the patient-provider relationship and the quality of care received, but only if it is done in the context of confidentiality and trust. Thus, how the information about SU/MH is obtained may not matter as much to the patients as the ensuing conversation, which should be empathetic, supportive, and productive. Our next steps with this work are to pilot an intervention in which patients completing the PROs can select one result they would like to address with their provider, enhancing the patient-centeredness of the screening. Simultaneously, we will work to ensure that there are resources in place to help providers have supportive conversations and make appropriate treatment decisions and/or referral plans. We will emphasize the development of sustainable systems that do not excessively increase provider burden to have the largest potential impact on retention in HIV care.

## Additional file

Additional file 1: Interview Guides. Interview guides used for patient and provider interviews. (DOCX 20 kb)

## Abbreviations

ART: Antiretroviral therapy
ASSIST: Alcohol, Smoking and Substance Involvement Screening Test
AUDIT-C: Alcohol use disorders identification test
GAD-7: General anxiety disorder
HIV: Human immunodeficiency virus
JHHCC: Johns Hopkins Clinical Cohort
MH: Mental health
PHQ-8: Personal Health Questionnaire Depression Scale
PI: Principal investigator
PLWH: People living with HIV
PROs: Patient-reported outcomes
SU: Substance use
