# Supplementary material for: A qualitative study examining the benefits and challenges of incorporating patient-reported outcome substance use and mental health questionnaires into clinical practice to improve outcomes on the HIV care continuum
Source: BMC Health Serv Res. 2018 Jun 7;18:419. doi: 10.1186/s12913-018-3203-x (PMC5992635; doi:10.1186/s12913-018-3203-x)
Supplement: Supplementary file 1 — Interview Guides. Interview guides used for patient and provider interviews. (DOCX 20 kb) [file 12913_2018_3203_MOESM1_ESM.docx]

**Additional file 1. Interview Guides**

**Qualitative Interview Questions – Patients**

What questions, if any, do you have before we get started?

[Intro for people not retained in care]:

We see you have not been coming to visits with your doctor that often; we’re interested in hearing more about your experiences

[Intro for people retained in care]:

We’re interested in hearing about your experiences with care here at the XXX Clinic.

| Warm up question | 1. Tell me your thoughts about your care here at the XXX Clinic.   Probe  How many years have you been coming?  Was this your first clinic/where did you go previously?  Paint a picture for me of where you’ve gone for care previously.  What things do you like?  What things do you not like? |
| --- | --- |
| Retention | Now I’d like to ask you about some of the things that help you come to clinic visits.   1. What helps you keep your clinic visits? 2. What gets in the way of keeping your visits? 3. What more do you think the clinic could do to help you keep your visits? |
| Questionnaire Administration | Now I’d like to talk with you about the questionnaires that you complete on the computer when you come for a research visit.   1. When is the last time you came for a research visit? 2. What type of help do you get when taking the questionnaire? 3. Would you feel comfortable taking the questionnaire in a room with other patients?   Probe  If no, why not, what would you need to feel comfortable |
| MH/SU | Topics of the questionnaires include mental health issues, like depressive symptoms, and substance use (alcohol and drugs). You were selected to participate today because at some time in the past you reported depressive symptoms or substance use on the questionnaire.   1. What have you done when you needed help with mental health or substance use issues? 2. How have you and your doctor approached these issues? 3. Has your social worker ever referred you for treatment for mental health or drug/alcohol issues?   Probe  What happened when you got referred?   1. What worked best for you?   Probe For people in a similar situation, what do you think would work best for them?   1. What are your thoughts on how we could do things better when people need help with mental health or substance use issues?   Probe  What could the doctor do better?  What could the nurse do better?  What could the clinic do better? |
| Doctor – Patient | As a reminder, right now your doctor doesn’t see the results of the questionnaires, however, this may change.   1. What would be the advantages of your doctor getting the results of your questionnaire 2. What would be the disadvantages of your doctor getting the results of your questionnaire? 3. How do you think your visits with your doctor or provider would change if he or she had the answers to your questionnaire? 4. What is your opinion about documenting the questionnaire information in your chart in the future? |

**Qualitative Interview Questions – Clinic Provider**

What questions, if any, do you have before we get started?

| Today I’d like to cover several topics. The first is retention in HIV care. The second is the questionnaires that patients complete for research purposes, and your thoughts on transitioning those for clinical use.  So let’s get started.   1. In your opinion, what is the role of HIV clinicians in supporting retention in care? 2. What do you do already to try to increase retention? 3. Are you aware of any external programs that are working to increase retention in care?   *If yes:* What are your thoughts about these programs? What type of linkages back to the XXX Clinic do you think are needed?  Moving back to thinking about our clinic, as you may know,  Every 6 months, patients enrolled in the XXX Cohort are asked to complete a 15-20 minute survey. The survey includes validated questionnaires in the following domains:  -Depression/Anxiety (PHQ)  -Tobacco Use  -Alcohol Use (AUDIT-C with full AUDIT and MINI once a year if at-risk)  -Drug Use (ASSIST)  -Adherence to ART (AACTG, VAS, and self-rating item)  -Quality of Life (EuroQual; EQ-5D)  -Physical Activity (Lipid Research Clinics; LRCQ)  -Sexual risk behavior  Currently, a separate appointment is scheduled for the patient to complete the survey and it is used strictly for research. The survey is called the Patient-Reported Outcomes (or PRO) survey. We are considering changing the PROs from being used for research only to being used for clinical care. That would mean that you would have the PRO data at, or potentially after, your patient’s visit for use in the patient’s care.   1. How might the PROs be used to supplement the clinical interview?   Probes:  What is potentially gained from using the PRO to supplement the clinical interview?  What is potentially lost from using the PRO to supplement the clinical interview?  One thing that we know affects retention is unrecognized or untreated mental health and substance use issues. We may potentially increase recognition of mental health and substance use issues using the PROs.   1. How do you currently address substance use issues you detect in clinical encounters?   Probes:  Do you feel that your current practice is effective for getting patients the care they need?  What would be added with the PRO data?   1. What would we need to do so that you can use the PRO data?   Probes: How would you like (or how do you anticipate) positive screens for substance use or mental health issues would be managed in your clinic?  With regards to the other domains (adherence to ART, physical activity, sexual risk behavior)   1. What is potentially gained from using the PRO to supplement the clinical interview? 2. What is potentially lost from using the PRO to supplement the clinical interview? 3. What would we need to do so that you can use the PRO data? |
| --- |
| Moving away from the domains into a couple of general questions,   1. In terms of the results themselves, would you like them as a paper or entered into the electronic medical record? 2. Would you like to receive the (score category/interpretation) or the (score category/interpretation plus a recommended action based on the score category)? |

That concludes our interview.

Thank you very much for your time. If you have any questions, please feel free to contact the study team.
